# Exploring Peacebuilding Strategies to Develop Teacher-Student Interpersonal Relationships in English as a Foreign Language/English as a Second Language Classrooms

**DOI:** 10.3389/fpsyg.2021.736315

**Published:** 2021-08-12

**Authors:** Jiangtao Fu

**Affiliations:** ^1^School of Foreign Languages, Henan University, Kaifeng, China; ^2^English Language and Literature Research Center, Henan University, Kaifeng, China

**Keywords:** English as a foreign language/English as a second language classrooms, interpersonal relationships, peacebuilding, positive psychology, teacher-trainers

## Abstract

In the conflict-affected era, there is now an urgent need for a peaceful world. Although the relevance of peace in language education, within English as a second language (ESL) or English as a foreign language (EFL), may seem irrelevant to some, the language of peace utilizes an interdisciplinary method that supports students in creating more reasonable discussions. Alternatively, the attention of language teaching is just on the development of cognition in preference to emotions, whereas methods that sustain the theory of the whole person through positive psychology should be presupposed. This review seeks to explore the connection between multiple dimensions of peace and the certain strategies and activities that can be implemented to build peace in EFL/ESL classrooms. Further, the related strategies on the issues, such as self-regulation, engagement, mindfulness, and motivations, are proposed. In a nutshell, the implications of peacebuilding for teachers, teacher-trainers, and future researchers are presented, and new directions for future research are set out.

## Introduction

People around the world have confronted with diverse kinds of violence and conflicts derived from some sources, and such violence and conflicts may affect them in a negative way (Agnihotri, 2017); hence, one way to react against violence and its negative effects is peace education that can be presented through personal, interpersonal, and ecological peace (Snauwaert, 2020). As Malala Yousafzai, a Nobel Peace Prize winner, stated, education was the means through which peace could be achieved (Falk, 2013). Peace is a permanent progression that has four components, such as an outcome, a process through negotiation, an individual character, and a culture (Leckman et al., 2014). The whole world seriously needs peace at multiple levels, and the global role of English spotlights its dimensions to bring people together through language peace (Gkonou et al., 2021). Also, language is fundamental to strategies for keeping, making, and building peace (Oxford, 2014). Among the elements for such peace, positive communication is the key figure known as the language of peace that facilitates agreement in multiple dimensions *via* both verbal and nonverbal communications (Oxford, 2017). A positive feature of peace makes its validation a matter of choice, representing a way of life where one decides on the growth of other’s wellbeing that has been accentuated by positive psychology (PP) whose purpose is to approve ways of living that are pleasant (Seligman, 2018), and bring about a life worth living (Csikszentmihalyi and Csikszentmihalyi, 2006).

Peacebuilding, which emerged in peace studies and the work of Galtung (1996), is considered one of the active social routes within language education that strives for creating viable and practical peace through the transformative process that indicates a constant process of altering relations, manners, and attitudes from the negative to the positive (Oxford et al., 2020). When positivity is added to peace, the idea of peace reveals what is taking place in a particular situation (Gregersen and MacIntyre, 2021). Peace psychology, therefore, underscores peace through a nonviolent approach to promote proper actions. This is the same when the word positive goes with psychology that shifts the learners’ point of view from oneself toward improving their minds and performance through their activities (Gregersen and MacIntyre, 2021).

However, education also has a negative aspect that can alleviate conflict or build peace (Bush and Saltarelli, 2000). Peace and conflict happen within each other, in different parts, across time and settings. So, a peacebuilder might be supposed to provide more chances for positive engagements which subsequently may diminish the rate of conflicts. This is congruent with Peterson (2006) who declared that PP is the study of the factors affecting life worth living, not just the elimination or exclusion of the challenges. Even though, at the first step, when talking about peace, the words war, conflict, aggression, and inequality come to a person’s mind, the priority based on PP should be given to how enthusiastically build peace and social justice instead of focusing merely on how to avoid or abolish violence (Gibson, 2011).

Peacebuilding education requires discussion across variations for comprehending and controlling conflicts by cultivating positive relations, reassuring social schemes, and coordination between groups and cultures (Olivero and Oxford, 2019). To provide peacebuilders with both theoretical and practical ideas, applicable strategies should be taken into valuable consideration to be integrated into their language teaching settings. It is assumed that emotions affect the strategies learners select to use; as a result, it affects their levels of engagement, learning, and achievement too (Pekrun and Linnenbrink-Garcia, 2012).

Despite the collection of studies and reviews in this field, to the best of the researcher’s knowledge, there is not enough evidence that focuses on peacebuilding along with its strategies and activities through the theory of PP in the EFL/ESL classrooms. As an effort to fill this research gap, the current minireview contemplates first the multidimensional peace and accordingly its related strategies and activities with the focus on interpersonal peace that is in line with PP.

## Multidimensional Peace

There are different dimensions in Oxford’s (2013) model for promoting multidimensional peace through language learning. The first one which is the core of the other dimensions of peace is the inner peace, powerfully determined by learners’ self-concept and individual attributes (Amerstorfer, 2021). The second type of peace is interpersonal which relies on people’s assertiveness, behaviors, and skills (Waelde et al., 2017). The third one is the intergroup peace dimension that entails preserving peace between groups of people according to their gender, culture, religion, and race (Gabryś-Barker, 2012). The next one, intercultural peace refers to congruence among the world; each of them views itself as cohesive by the public (Boulding, 2000). The fifth is international peace that implies achieving peace among countries around the world which encompasses realization further than constricted wellbeing and move toward global wellbeing (Oxford, 2020). The last one is ecological peace which talks about being cautious about the environment and its whole species (Oxford and Lin, 2011).

### Strategies and Activities of Multidimensional Peace

Thanks to the internal causes of stress, inner peace can be endangered (Oxford, 2017). Therefore, by utilizing the features of PP and assumption of the peace approach, teachers should strive to detect ways to adjust their negative emotions and strengthen positivity that can be enhanced through relevant and appropriate strategies (Barbeito and Sánchez Centeno, 2018).

These strategies are tied up with learners’ self-regulation, motivation, autonomy, mindsets, self-efficacy, resilience, and internal attributions for achievement, displaying the complexity of EFL/ESL learning (Oxford, 2017). One of these strategies is self-regulation that signifies the learners’ capability of monitoring their learning, and it can be feasible by negotiation with the more skilled person through scaffolding, and it is classified by stimulating a goal, selecting and using relevant strategies (Oxford, 2011). Learning to regulate emotional stress and taking part in critical practice can help teachers to deal with stressful situations which per se enhances their learners’ achievement (Fathi et al., 2020).

The other issues that supported interpersonal peace include empathy, positive revision of conflict circumstances, tolerance, and mindfulness (Rizkalla et al., 2008; Waelde et al., 2017). In their research of Rizkalla et al. (2008) evinced that people who are not able to sympathize with others are more expected to be involved in the conflict.

The use of mindfulness throughout interpersonal conflict enhanced regulation with negative behaviors by others, and in the conflict, those who are more mindful revealed better stress responses (Laurent et al., 2016). In a research carried out by Alkoby et al. (2017), individuals who were exposed to mindfulness experienced the decline of their negative emotions and perception regarding those they conflict with, and the portability of mindfulness into daily tasks can enhance engagement in peacebuilding dialogue.

The realization of interpersonal and intergroup peace entails the advancement of negotiation and conflict resolution strategies that raise empathy between people and lead to the absence of violence. Indeed, these types of activities pave the ways for both teachers and learners to be engaged in conflictual issues in subject matter that help teachers to elicit the contrasting perspectives of diverse students, and through this teacher-student interpersonal relationships, their positive emotion arises that it has gained remarkable popularity in academic research (Mystkowska-Wiertelak, 2020). This is in line with Hiver et al.’s (2021) postulation, declaring that L2 engagement happens when a language learner is spiritually or physically engaged in the process of doing tasks.

Furthermore, through interpersonal activities, such as lectures, discussions, and cooperative group works, opportunities are provided for learners to become critical thinkers, and also develop their mindful attitude, as well (Miller et al., 2019). As Waelde et al. (2017) pinpointed, through Interpersonal activities, learners are tolerant of opposing viewpoints and have interest-based discussions which also boost their motivation and correspondingly result in their overall fulfillment (Olivero & Oxford, 2019). Several studies proved the relationship between teachers’ interpersonal communication behaviors and students’ engagement, motivation, and success (Derakhshan, 2021; Xie and Derakhshan, 2021).

Additionally, to cultivate skills in intercultural communication, various constructs, namely, mindfulness, critical thinking, metacognition, cognitive flexibility, cultural flexibility, and intercultural empathy, are at the center of attention (Wei and Zhou, 2021). EFL/ESL students are supposed to learn strategies to clear up the conflicts that are associated with racism, unfair behavior, bias, and confusion. Besides, ecological peace can be supported by some activities that aimed to help students care about nature, either through verbal or nonverbal forms of language (Oxford, 2020).

## Implications and Future Directions

Since the focus of PP was on wellbeing and personal resources for resilience along with intrapersonal peace, it inclines to neglect probable negative significances for others in the broader social setting, and to date, PP has said little about how it might be settled to foster social justice and the wellbeing of people who face oppression, within nations as well as globally (Becker and Marecek, 2008). Therefore, by assisting expected teachers to turn into more reflective and purposeful peacebuilders, teacher educators can integrate positive peace tasks into their syllabi. Teachers are agents in peacebuilding employing pedagogy and programs to tackle discrimination and conflict (Horner et al., 2015). In these types of changes, multiple dimensions of positive peace can stimulate innovative ways of thinking about self and others.

The contributions of this review increase consciousness of the importance of encouraging peace in the process of language education and agreed on some beliefs about peace, such as its positivity that is incongruent with PP tasks that prepare situations in which peace flourishes. Through these pedagogical interferences, learners and teachers are suggested to regulate their emotions and be more confident. Indeed, through some interpersonal strategies, such as empathy that can be used efficiently in intergroup programs individuals can realize the emotions, thoughts, and perspectives of people from other groups much better that results in conflict reductions. On the whole, to promote peace, EFL/ESL must integrate the progress of learners’ critical thinking to convey meaning through the presence of many communicative activities, such as discussions, role-plays, pair work, and problem-solving activities (Kruger, 2012). However, the tasks can be re-designed by teachers in various circumstances grounded on the personality types of their students and their culture in order to motivate them to be involved.

Teachers are persuaded to employ types of activities that facilitate multidimensional peace in their classrooms. However, these activities should be examined again in further studies to show in what way they function with diverse types of people in various situations. Inner peace makes teachers powerful to be inclined to utilize emotional self-regulation in learners to create peace at the primary stages of instruction. Activities that manifest inner peace can help teachers lessen negative emotions and increase positive ones leading to wellbeing in the classroom. Due to learners’ stress or lack of self-confidence in addition to social tensions and pressures, a safe setting is worthwhile for the inner peace of learners along with the interpersonal peace that comes true by collaboration. EFL/ESL learners who collaborate in teams should respect and trust each other which are possible through self-regulation, engagement, and teamwork (Amerstorfer, 2021). However, it is also suggested to shift the focus onto specific types of tasks and activities to check out the incorporation of intercultural and ecological peace in EFL/ESL learning.

## Author Contributions

The author confirms being the sole contributor of this work and has approved it for publication.

## Conflict of Interest

The author declares that the research was conducted in the absence of any commercial or financial relationships that could be construed as a potential conflict of interest.

## Publisher’s Note

All claims expressed in this article are solely those of the authors and do not necessarily represent those of their affiliated organizations, or those of the publisher, the editors and the reviewers. Any product that may be evaluated in this article, or claim that may be made by its manufacturer, is not guaranteed or endorsed by the publisher.
